# 3C-like protease inhibitors block coronavirus replication in vitro and improve survival in MERS-CoV–infected mice

**DOI:** 10.1126/scitranslmed.abc5332

**Published:** 2020-08-19

**Authors:** Athri D. Rathnayake, Jian Zheng, Yunjeong Kim, Krishani Dinali Perera, Samantha Mackin, David K. Meyerholz, Maithri M. Kashipathy, Kevin P. Battaile, Scott Lovell, Stanley Perlman, William C. Groutas, Kyeong-Ok Chang

**Affiliations:** 1 Department of Chemistry, Wichita State University, Wichita, KS 67260, USA.; 2 Department of Microbiology and Immunology, University of Iowa, Iowa City, IA 52242, USA.; 3 Department of Diagnostic Medicine and Pathobiology, College of Veterinary Medicine, Kansas State University, Manhattan, KS 66506, USA.; 4 Department of Pathology, University of Iowa, Iowa City, IA 52242, USA.; 5 Protein Structure Laboratory, University of Kansas, Lawrence, KS 66045, USA.; 6 NYX, New York Structural Biology Center, Upton, NY 11973, USA.

## Abstract

Pathogenic coronaviruses are a major threat to global public health, as exemplified by severe acute respiratory syndrome coronavirus (SARS-CoV), Middle East respiratory syndrome coronavirus (MERS-CoV), and the newly emerged SARS-CoV-2, the causative agent of coronavirus disease 2019 (COVID-19). We describe herein the structure-guided optimization of a series of inhibitors of the coronavirus 3C-like protease (3CLpro), an enzyme essential for viral replication. The optimized compounds were effective against several human coronaviruses including MERS-CoV, SARS-CoV, and SARS-CoV-2 in an enzyme assay and in cell-based assays using Huh-7 and Vero E6 cell lines. Two selected compounds showed antiviral effects against SARS-CoV-2 in cultured primary human airway epithelial cells. In a mouse model of MERS-CoV infection, administration of a lead compound 1 day after virus infection increased survival from 0 to 100% and reduced lung viral titers and lung histopathology. These results suggest that this series of compounds has the potential to be developed further as antiviral drugs against human coronaviruses.

## Targeting coronavirus 3CLpro

Coronavirus 3C-like proteases (3CLpro) are attractive therapeutic targets because they play a vital role in coronavirus replication. Rathnayake *et al.* now report a series of optimized coronavirus 3CLpro inhibitors that blocked replication of the human coronaviruses MERS-CoV and SARS-CoV-2 in cultured cells. Administration of a lead compound to a MERS-CoV mouse model demonstrated proof-of-concept efficacy. These findings suggest that this lead compound should be investigated further as a potential therapeutic for human coronavirus infection.

## Teaser

Optimized small-molecule 3C-like protease inhibitors show potency against human coronaviruses in both cell culture and a mouse model.

## INTRODUCTION

Coronaviruses are a large group of viruses that can cause a wide variety of diseases in humans and animals (*1*). Human coronaviruses generally cause the common cold, a mild upper respiratory illness. However, global outbreaks of new human coronavirus infections with severe respiratory disease have periodically emerged from animal reservoirs, including severe acute respiratory syndrome coronavirus (SARS-CoV), Middle East respiratory syndrome coronavirus (MERS-CoV), and, most recently, SARS-CoV-2, the causative agent of coronavirus disease 2019 (COVID-19). SARS-CoV-2 emerged in China in December 2019 and subsequently rapidly spread throughout the world. Genetic analysis of SARS-CoV-2 revealed that it is closely related to SARS-like betacoronaviruses of bat origin, bat-SL-CoVZC45 and bat-SL-CoVZXC21 (*2*). Despite the periodic emergence of new coronaviruses capable of infecting humans, there are currently no licensed vaccines or antiviral drugs against any coronaviruses, underscoring the urgent need for the development of preventive and therapeutic measures against pathogenic coronaviruses.

The coronavirus genome contains two overlapping open reading frames (ORF1a and ORF1b) at the 5′ end terminal, which encode polyproteins pp1a and pp1ab. The polyproteins are processed by a 3C-like protease [3CLpro or main protease (MPro)] (11 cleavage sites) and a papain-like protease (PLpro) (3 cleavage sites), resulting in 16 mature nonstructural proteins, including an RNA-dependent RNA polymerase (RdRp). Both 3CLpro and PLpro are essential for viral replication, making them attractive targets for drug development (*3*–*7*). Coronavirus 3CLpro is a cysteine protease that has two N-terminal domains containing two β-barrel chymotrypsin-like folds (*8*–*10*). The active site of 3CLpro is located in the cleft between the two domains and is characterized by a catalytic Cys-His dyad.

We have developed broad-spectrum inhibitors of an array of viruses, including coronaviruses and noroviruses (*11*–*18*) that use 3CLpro for viral replication and picornaviruses that use 3C protease (*19*). We have shown efficacy of the coronavirus 3CLpro inhibitor GC376 (currently in clinical development) in animal models of coronavirus infection (*20*, *21*). Specifically, administration of GC376 to cats with feline infectious peritonitis (FIP), a coronavirus-induced systemic disease that is 100% fatal, reversed the progression of FIP and resulted in clinical remission (*20*, *21*). We have recently reported the results of exploratory in vitro studies using a dipeptidyl series of MERS-CoV 3CLpro inhibitors that embody a piperidine moiety as a new design element, as well as pertinent structural and biochemical studies (*17*). Here, we report the development of 3CLpro inhibitors against multiple coronaviruses, including SARS-CoV-2, and demonstrate in vivo efficacy against MERS-CoV in a mouse model.

## RESULTS

### 3CLpro inhibitors show activity against multiple coronaviruses in enzyme and cell-based assays

**Fig. 1:**
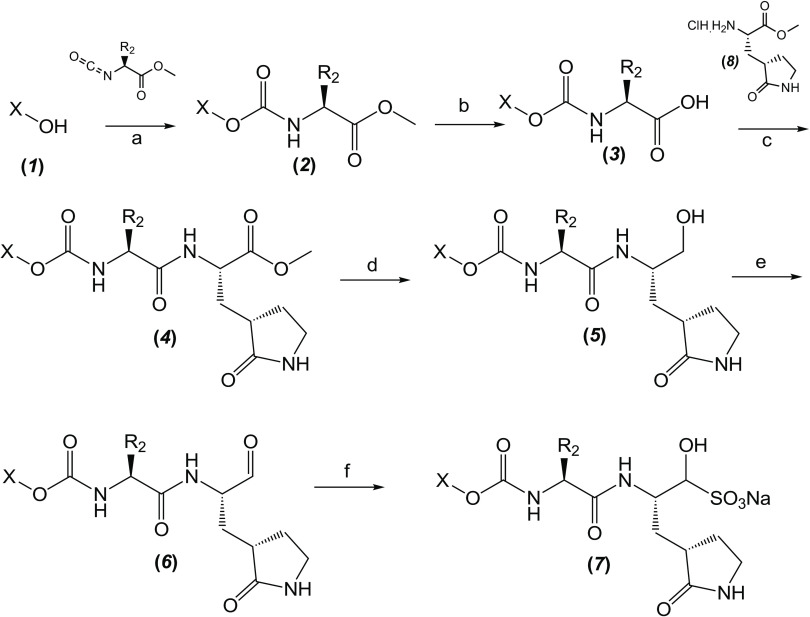
Synthesis scheme for compound series *6a* to *6k* and *7a* to *7k*. Stepwise compound synthesis with intermediate compounds is shown for 3C-like protease (3CLpro) inhibitors of the *6a* to *6k* and *7a* to *7k* series. The alcohol inputs were reacted with (L) leucine isocyanate methyl ester or (L) cyclohexylalanine isocyanate methyl ester to yield products, which were then hydrolyzed to the corresponding acids with lithium hydroxide in aqueous tetrahydrofuran. Subsequent coupling of the acids to glutamine surrogate methyl ester “***8***” furnished compounds “***4***”. Lithium borohydride reduction yielded alcohols “***5***”, which were then oxidized to the corresponding aldehydes “***6***” with Dess-Martin periodinane reagent. The bisulfite adducts “***7***” were generated by treatment with sodium bisulfite in aqueous ethanol and ethyl acetate. ^a^Amino acid methyl ester isocyanate/TEA/CH_3_CN/reflux/2 hours; ^b^1M LiOH/THF/RT/3 hours; ^c^EDCI/HOBT/glutamine surrogate/DIPEA/DMF/RT/24 hours; ^d^2M LiBH_4_/THF/methanol/RT/12 hours; ^e^Dess-Martin periodinane/DCM/15° to 18°C/3 hours; ^f^NaHSO_3_/ethyl acetated/ethanol/H_2_O/44° to 55°C. Full details are provided in Supplementary Materials and Methods.

The synthesis scheme for compound series ***6a*** to ***6k*** and ***7a*** to ***7k*** is shown in Fig. 1 and described in Supplementary Materials and Methods. The activity of compounds ***6a*** to ***6k*** and ***7a*** to ***7k*** against the 3CLpro enzymes of MERS-CoV, SARS-CoV, and SARS-CoV-2 was evaluated in a fluorescence resonance energy transfer (FRET) enzyme assay (Table 1 and table S1). Several compounds in this series (***6a***, ***7a***, ***6c***, ***7c***, ***6e***, ***7e***, ***6h***, ***7h***, ***6j***, and ***7j***) were also tested in cell-based assays (Table 2). Table 1 and table S1 show 50% inhibitory concentration (IC_50_) values in a FRET enzyme assay for select compounds (***6a***, ***7a***, ***6c***, ***7c***, ***6e***, ***7e***, ***6h***, ***7h***, ***6j***, and ***7j***) and GC376. Fifty percent inhibitory concentration (EC_50_) values and 50% cytotoxic concentration (CC_50_) values for select compounds and GC376 were measured in cell culture assays (Table 2). Cell culture assays included Huh-7 cells infected with MERS-CoV, Vero E6 cells infected with SARS-CoV-2, the Crandell-Rees Feline Kidney (CRFK) cells infected with FIP virus (FIPV), and L929 cells infected with mouse hepatitis virus (MHV) (Table 2). Inhibitors with a P_2_ leucine (Leu) residue were more potent than those with a cyclohexylalanine against MERS-CoV 3CLpro (compounds ***6h*** and ***7h*** versus ***6i*** and ***7i***), with submicromolar IC_50_ values (Table 1 and table S1). The compounds tested against MERS-CoV in cell culture (***7a***, ***6c***, ***7e***, ***7h***, and ***6j***) also displayed submicromolar EC_50_ values. Among these compounds, ***6j*** showed the most potent antiviral activity against MERS-CoV with an EC_50_ value of 0.04 μM. GC376 with a P_2_ Leu residue and a nonfluorinated benzyl cap exhibited 20-fold lower potency against MERS-CoV in cell culture compared to compound ***6j*** (Table 2).

**Table 1:** Structures of 3CLpro inhibitors and their IC_50_ values in the FRET enzyme assay. IC_50_, 50% inhibitory concentration; FRET, fluorescence resonance energy transfer. 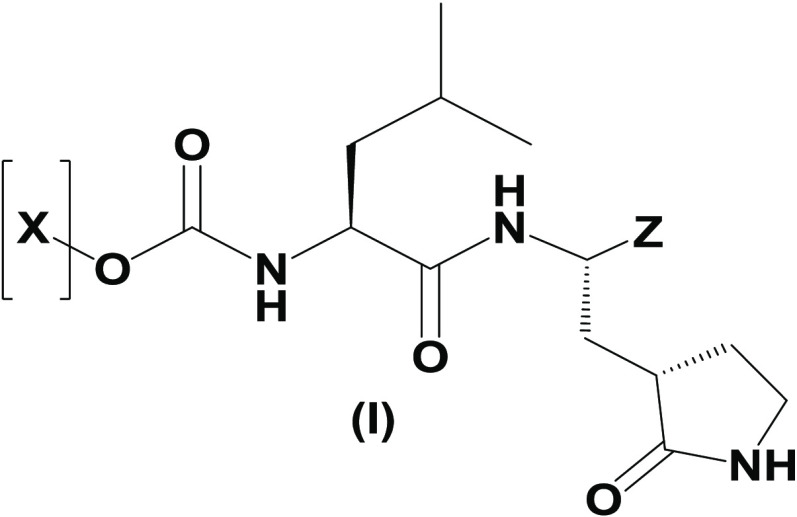

**Compound**	**[X]**	**Z**	**IC_50_ in enzyme assay (μM)**		
			**MERS-CoV**	**SARS-CoV**	**SARS-CoV-2**
*6a*	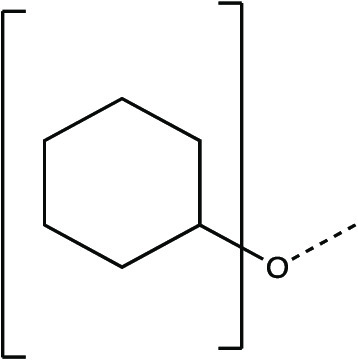	CHO	0.35 ± 0.1	3.9 ± 0.3	0.82 ± 0.1
*7a*		CH(OH)SO_3_Na	0.15 ± 0.01	3.8 ± 0.4	0.65 ± 0.06
*6c*	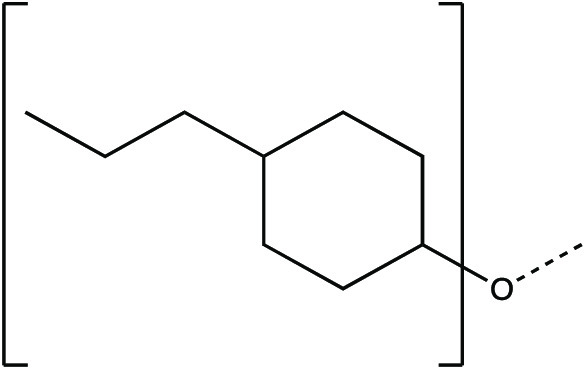	CHO	0.15 ± 0.06	1.7 ± 0.2	0.28 ± 0.05
*7c*		CH(OH)SO_3_Na	0.10 ± 0.04	1.7 ± 0.1	0.23 ± 0.05
*6e*	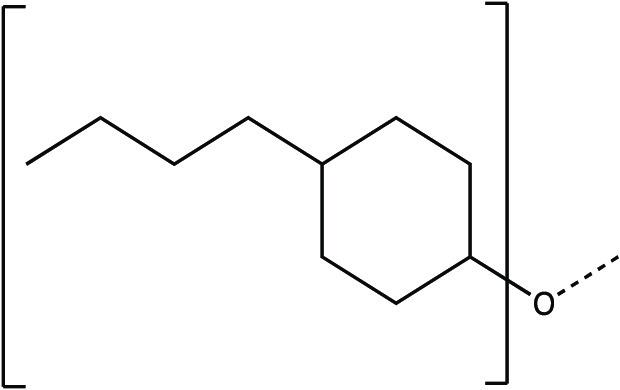	CHO	0.15 ± 0.02	0.9 ± 0.1	0.17 ± 0.06
*7e*		CH(OH)SO_3_Na	0.13 ± 0.06	1.0 ± 0.1	0.20 ± 0.06
*6h*	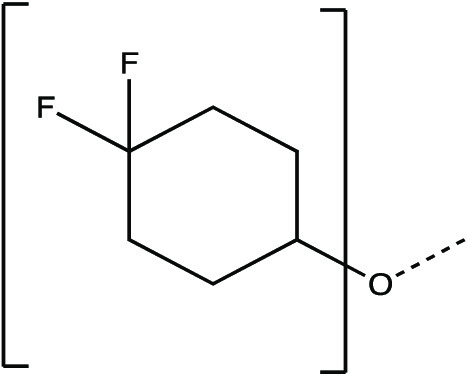	CHO	0.07 ± 0.01	2.3 ± 0.3	0.43 ± 0.1
*7h*		CH(OH)SO_3_Na	0.08 ± 0.01	2.2 ± 0.1	0.41 ± 0.1
*6j*	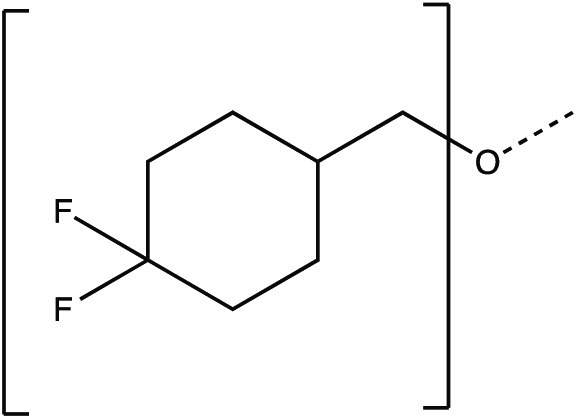	CHO	0.08 ± 0.01	1.2 ± 0.2	0.48 ± 0.08
*7j*		CH(OH)SO_3_Na	0.1 ± 0.01	1.1 ± 0.1	0.45 ± 0.1
***GC376***	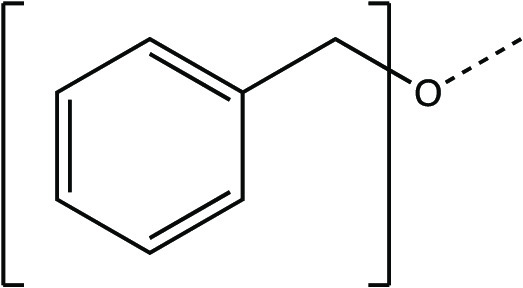	CH(OH)SO_3_Na	0.41 ± 0.2	2.2 ± 0.1	0.62 ± 0.08

**Table 2:** 3CLpro inhibitors and their EC_50_ and CC_50_ values in cell culture assays. CC_50_, 50% cytotoxic concentration; MHV, mouse hepatitis virus; FIPV, feline infectious peritonitis.

**Compound**	**EC_50_ in cell culture (μM)**				**CC_50_ (μM)**
	**MERS-CoV**	**SARS-CoV-2**	**MHV**	**FIPV**	
*6a*	ND*	ND	0.12 ± 0.05	0.09 ± 0.02	>100
*7a*	0.08 ± 0.1	ND	0.08 ± 0.03	0.07 ± 0.02	>100
*6c*	0.05 ± 0.06	0.25 ± 0.15	0.13 ± 0.05	0.12 ± 0.01	>100
*7c*	ND	ND	0.16 ± 0.08	0.17 ± 0.03	>100
*6e*	ND	0.15 ± 0.14	0.12 ± 0.04	0.22 ± 0.03	63.3 ± 2.3
*7e*	0.53 ± 0.2	ND	0.13 ± 0.02	0.17 ± 0.02	59.1 ± 3.5
*6h*	ND	0.9 ± 0.8	0.08 ± 0.05	0.12 ± 0.05	>100
*7h*	0.21 ± 0.1	ND	0.11 ± 0.08	0.12 ± 0.06	>100
*6j*	0.04 ± 0.02	0.8 ± 0.7	0.20 ± 0.07	0.08 ± 0.02	>100
*7j*	ND	ND	0.16 ± 0.05	0.07 ± 0.01	>100
GC376^#^	0.9 ± 0.2	ND	1.1**	0.04**	>100

*ND, not determined; the bisulfite adduct [CH(OH)SO_3_Na] reverted to the aldehyde (CHO) form, and so, only one form was examined in the cell-based assay.

**Previously reported (*13*, *19*).

The compounds were also effective against SARS-CoV-2 with EC_50_ values ranging from 0.15 to 0.9 μM in Vero E6 cells (Table 2). These compounds were also found to be potent against FIPV and MHV, with EC_50_ values ranging from 0.07 to 0.22 μM. In the FRET enzyme assay, these compounds were active against the 3CLpro of SARS-CoV and SARS-CoV-2 (Table 1). The IC_50_ values of these compounds against SARS-CoV-2 3CLpro ranged from 0.17 to 0.82 μM. Among these compounds, ***6e*** showed the most potent antiviral activity against SARS-CoV-2 in the enzyme assay (IC_50_, 0.17 μM) and cell-based assay (IC_50_, 0.15 μM) (Tables 1 and 2). GC376 also exhibited activity against the 3CLpro of SARS-CoV-2 with an IC_50_ value of 0.62 μM in the enzyme assay (Table 1).

The antiviral effects of compounds ***6j*** and ***6e*** against SARS-CoV-2 were confirmed in cultured primary human airway epithelial cells from three donors. In the absence of a 3CLpro inhibitor, viral titers in the infected cultured primary human airway epithelial cells reached 10^7.3^ (donor 1), 10^7.1^ (donor 2), and 10^8.4^ (donor 3) plaque-forming units (PFU) per milliliter of culture medium. In the presence of compound concentrations that were about two- to threefold higher than the EC_50_ values obtained in Vero E6 cells (2 μM, ***6j*** or 0.5 μM, ***6e***), viral titers were reduced to 10^6.4^ (donor 1), 10^6.1^ (donor 2), and 10^6.3^ (donor 3) PFU/ml for compound ***6j*** or 10^6.1^ (donor 1), 10^6.5^ (donor 2), and 10^8.1^ (donor 3) PFU/ml for compound ***6e*** (Table 3). Although there was some variation in viral replication among infected cells from the three donors (especially donor 3), the antiviral effects of both ***6j*** and ***6e*** were evident at the tested concentrations. For infected cells from donors 1 and 2, both compounds inhibited viral replication about 10-fold at the tested concentrations. For infected cells from donor 3, ***6e*** inhibited virus replication about 50%, whereas ***6j*** inhibited virus replication 100-fold at the tested concentrations.

**Table 3:** Antiviral effects of compounds *6j* and *6e* against SARS-CoV-2 in cultured primary human airway epithelial cells. The study was a single experiment, and viral titers were measured in duplicate with a plaque-forming assay.

**Compound** **(concentration)**	**Donor number**	**Virus titers (log_10_)**
Vehicle control	1	7.29 ± 0.05
	2	7.14 ± 0.02
	3	8.44 ± 0.06
*6j* (2.0 μM)	1	6.35 ± 0.02
	2	6.10 ± 0.09
	3	6.30 ± 0.1
*6e* (0.5 μM)	1	6.12 ± 0.01
	2	6.50 ± 0.01
	3	8.08 ± 0.01

### Cocrystal structures for 3CLpro of MERS-CoV, SARS-CoV, and SARS-CoV-2 with 3CLpro inhibitors

**Fig. 2:**
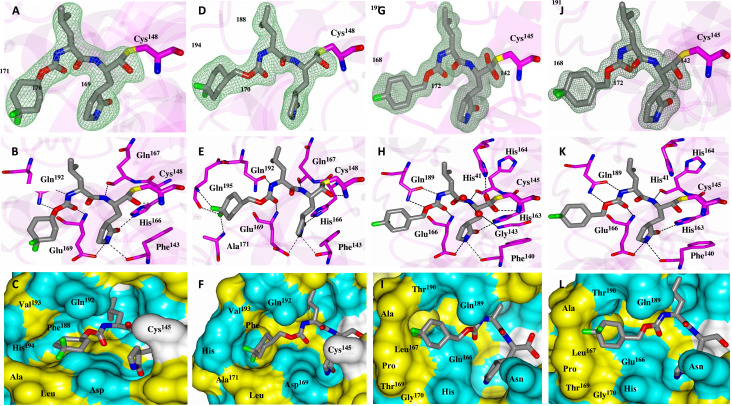
X-ray cocrystal structures of compounds with coronavirus 3CL proteases. Shown are cocrystal structures of the Middle East respiratory syndrome coronavirus (MERS-CoV) 3CLpro with compound ***6h*** (**A** to **C**) and MERS-CoV 3CLpro with compound ***7j*** (**D** to **F**). Shown are cocrystal structures of the severe acute respiratory syndrome coronavirus (SARS-CoV) 3CLpro (**G** to **I**) or SARS-CoV-2 3CLpro (**J** to **L**) with compound ***7j***. (A), (D), (G), and (J) show *F*_o_-*F*_c_ omit maps (green mesh) contoured at 3σ. (B), (E), (H), and (K) show hydrogen bond interactions (dashed lines) between the inhibitor and the 3CL protease. (C), (F), (I), and (L) show electrostatic surface representation of the binding pocket occupied by the inhibitor. Neighboring residues are colored yellow (nonpolar), cyan (polar), and white (weakly polar).

We determined multiple high-resolution cocrystal structures of compounds ***6b***, ***6d***, ***6g***, ***6h***, ***7i***, or ***7j*** with the 3CLpro of MERS-CoV (Fig. 2, A to F, and figs. S1 to S3). These inhibitors bound to the active site of MERS-CoV 3CLpro, demonstrating that the vicinity of the S_4_ pocket is encompassed by an array of primarily hydrophobic residues, including Phe^188^, Val^193^, Ala^171^, and Leu^170^ (Fig. 2, C and F, and fig. S3). Hydrophobic and hydrogen-bonding functionalities were incorporated into the 3CLpro inhibitors to capture additional interactions, and the position of the cyclohexyl moiety was also examined using appropriate congeners. The bisulfite adducts reverted to the corresponding aldehydes, which subsequently reacted with Cys^148^ to form nearly identical covalent complexes with a tetrahedral arrangement at the newly formed stereocenter (Fig. 2, A to F, and figs. S1 to S3). The backbone of compound ***6h*** (Table 1 and Fig. 2, A to C) engaged in H-bond interactions with amino acid residues Gln^192^, Gln^167^, and Glu^169^. Three additional side-chain H-bonds between the γ-lactam ring and His^166^, Phe^143^, and Glu^169^ also were clearly evident (Fig. 2B). Furthermore, the side chain of the P_2_ Leu was ensconced in the hydrophobic S_2_ pocket (Fig. 2C). The extra methylene group in compound ***7j***, which was converted to an aldehyde and thus became identical to ***6j***, resulted in the reorientation of the difluorocyclohexyl group and the formation of three H-bonds between Gln^195^ and Ala^171^ and the fluorine atoms, with concomitant loss of one of the Gln^192^ hydrogen bonds and the displacement of Phe^143^ (Fig. 2E). The substitution of the P_2_ Leu with P_2_ cyclohexylalanine (compound ***7i***; table S1) resulted in the loss of an H-bond with Gln^192^ but otherwise adopted the same interactions as observed for compound ***6h*** (fig. S2A). The electron density, hydrogen bond interactions, and electrostatic surface representations for MERS-CoV 3CLpro in complex with compounds ***6b***, ***6g***, and ***6d*** are shown in figs. S1 to S3.

Next, compound ***7j*** was cocrystallized with SARS-CoV or SARS-CoV-2 3CLpro, and the structures were compared to that for MERS-CoV 3CLpro and ***7j***. In the SARS-CoV 3CLpro-***7j*** complex (Fig. 2, G to I), the backbone of compound ***7j*** formed direct H-bonds with Cys^145^, His^163^, His^164^, Glu^166^, and Gln^189^. Compound ***7j*** also formed an additional H-bond with His^41^ and a water-mediated contact with Gly^143^ (Fig. 2H). However, there was a loss of the three H-bonds between Gln^195^ and Ala^171^ and the fluorine atoms, compared to the cocrystal structure of ***7j*** with MERS-CoV 3CLpro. The electron density map was consistent with both possible enantiomers at the new stereocenter formed by covalent attachment of the Sγ atom of Cys^145^ in the cocrystal structure of SARS-CoV 3CLpro with ***7j***. The electron density map for compound ***7j*** in complex with SARS-CoV-2 3CLpro was most consistent with a single enantiomer, although it adopted a similar binding mode and hydrogen bond interactions as observed in the SARS-CoV 3CLpro-***7j*** cocrystal structure (Fig. 2, J to L). Superposition of compound ***7j*** with MERS-CoV 3CLpro, SARS-CoV 3CLpro, and SARS-CoV-2 3CLpro (fig. S4) revealed a very similar binding mode for ***7j*** among all three viral proteases.

### 3CLpro inhibitor treatment increases survival and recovery of body weight in infected hDPP4-KI mice

**Fig. 3:**
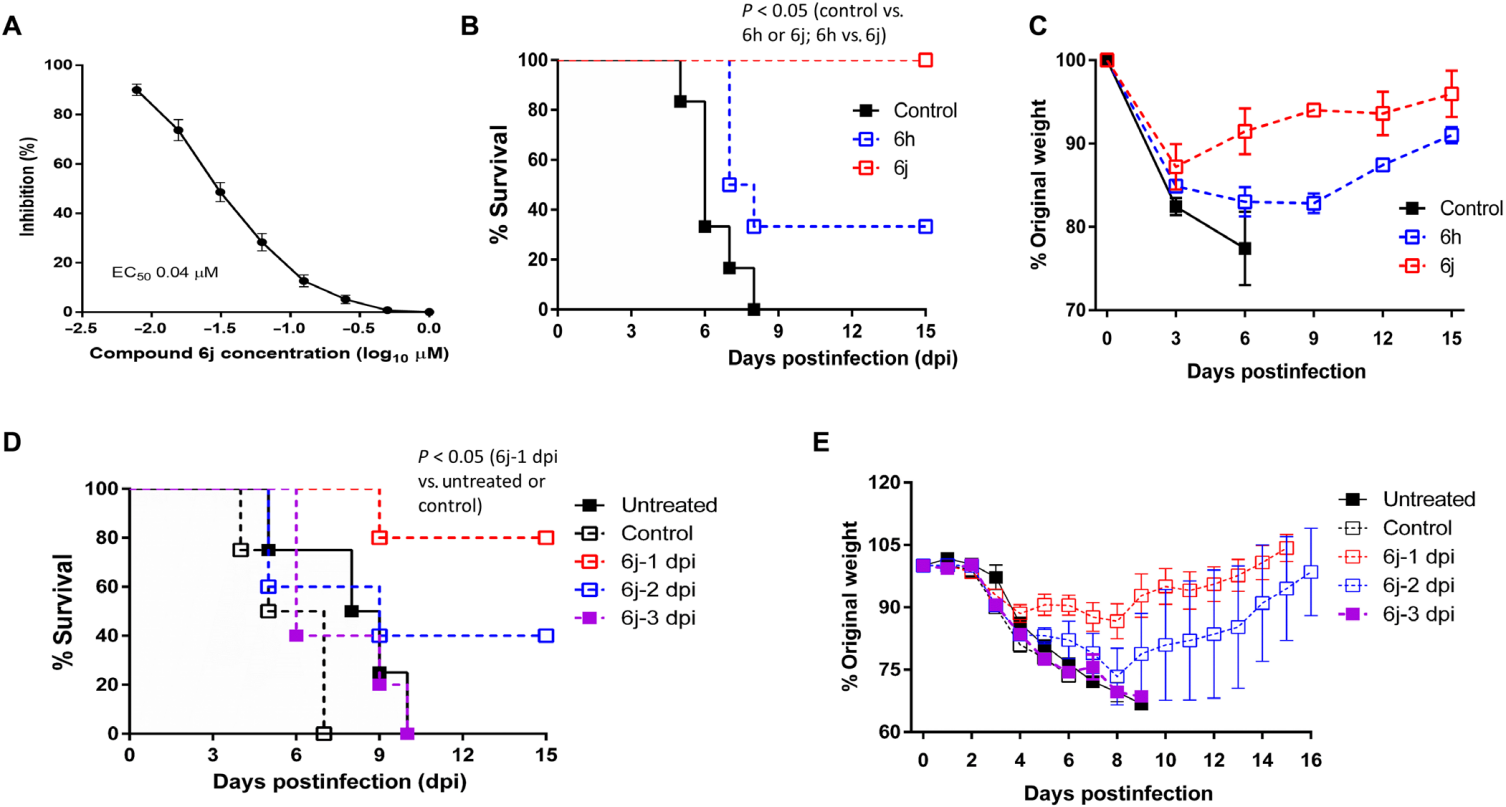
Effects of treating hDPP4-KI mice infected with MERS_MA_-CoV with compound *6j* or *6h*. (**A**) Shown is a dose-dependent curve for inhibition of MERS-CoV in cell culture by compound ***6j***. Serial dilutions of compound ***6j*** were added to confluent Huh-7 cells, which were immediately infected with MERS-CoV at a multiplicity of infection (MOI) of 0.01. After incubation of the cells at 37°C for 48 hours, viral titers were determined using a plaque-forming assay, and 50% inhibitory concentration (EC_50_) values were determined with GraphPad Prism software. (**B** and **C**) hDPP4-KI mice infected with mouse-adapted MERS-CoV (MERS_MA_-CoV) (*n* = 6) were treated with compound ***6j*** or ***6h*** starting at 1 day post virus infection (dpi) for up to 10 days, and survival (B) and body weight (C) were monitored for 15 days. Control mice received vehicle only. (**D** and **E**) hDPP4-KI mice infected with MERS_MA_-CoV were treated with compound ***6j*** (*n* = 5) starting at 1, 2, or 3 dpi, and survival (D) and body weight (E) were monitored for 15 days. Untreated mice and vehicle-treated mice (*n* = 4) were included as controls. Data points represent the mean and the SEM for one experiment. The analysis of survival curves in groups was performed using a log-rank (Mantel-Cox) test and a Gehan-Breslow-Wilcoxon test.

The most potent compound of the series, ***6j***, was identified in a cell-based assay and had an EC_50_ value of 0.04 μM against MERS-CoV (Fig. 3A). We determined the efficacy of compounds ***6j*** and ***6h*** in transgenic hDPP4-KI mice expressing human dipeptidylpeptidase 4, a model of MERS-CoV infection. First, hDPP4-KI mice were infected with the mouse-adapted MERS-CoV (MERS_MA_-CoV) virus strain and then were treated with compounds ***6h*** and ***6j*** (50 mg/kg per day, once a day) or vehicle as a control starting 1 day post virus infection (1 dpi) and continuing until 10 dpi. All mice treated with vehicle control died by 8 dpi (Fig. 3B). In contrast, 40% of mice treated with compound ***6h*** survived, and all mice treated with compound ***6j*** were alive at the end of the study (15 dpi) (Fig. 3B). The survival of mice treated with compound ***6j*** or ***6h*** was increased compared to the vehicle control (*P* < 0.05), and the ***6j***-treated mice had an improved survival rate compared to ***6h***-treated mice (*P* < 0.05). All mice treated with compound ***6j*** rapidly recovered from body weight loss starting at 3 dpi (Fig. 3C). The mice that survived after ***6h*** treatment continued to lose body weight until 6 dpi but then started to gain weight from 9 dpi (Fig. 3C).

After we observed that treatment with compound ***6j*** resulted in the survival of MERS_MA_-CoV–infected hDPP4-KI mice, we conducted another study by delaying treatment initiation until 3 dpi. Similar to the first study, no untreated mice or mice given vehicle (control) survived, and there was no statistical difference between these two groups (0% survival) (Fig. 3D). When ***6j*** treatment was started on 1 dpi, four of the five mice survived (80% survival), and there was a statistically significant increased survival in mice treated starting at 1 dpi compared to untreated or vehicle control–treated mice (*P* < 0.05; Fig. 3D). When ***6j*** treatment was delayed by one additional day (2 dpi), the survival of mice treated with ***6j*** decreased to 40%, but this was still higher than the 0% survival for untreated or vehicle control–treated mice. However, there was no statistical difference between the ***6j*** treatment group starting at 2 dpi and the untreated or vehicle control–treated groups (Fig. 3D). Treatment with ***6j*** starting at 3 dpi also failed to improve the survival of mice compared to the untreated or vehicle control–treated groups (Fig. 3D). All mice lost body weight after virus infection, but surviving mice treated with ***6j*** regained the lost weight by 15 dpi (Fig. 3E). Recovery of body weight was faster in mice treated with ***6j*** starting at 1 dpi than at 2 dpi (Fig. 3E). These results show that survival of mice markedly increased when ***6j*** was given at 1 dpi.

### Treating infected hDPP4-KI mice with compound *6j* reduces lung viral titers

**Fig. 4:**
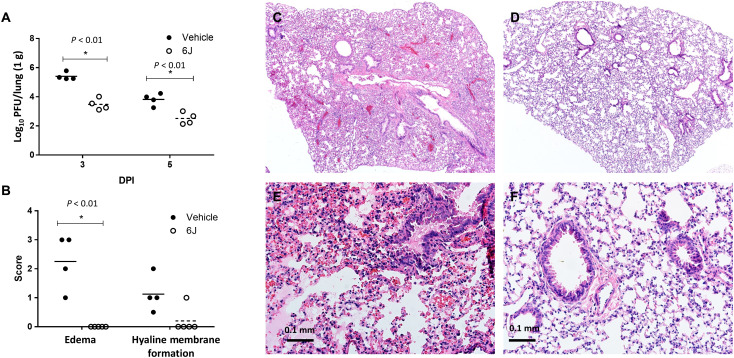
Lung virus titers and histopathology of *6j*-treated hDPP4-KI mice infected with MERS_MA_-CoV. hDPP4-KI mice were infected with MERS_MA_-CoV at 0 dpi and then were treated with vehicle as a control or with compound ***6j*** starting at 1 dpi until euthanasia (*n* = 4 or 5 per group). (**A**) Lungs were collected, and virus titers were measured at 3 and 5 dpi. Lungs were examined for edema and for hyaline membrane formation (**B**), and lung sections were stained with hematoxylin and eosin stain for histopathology at 6 dpi (**C** to **F**). (B) Tissues were scored for edema and hyaline membrane formation using the scale: 0, none; 1, rare (<5 alveoli); 2, <33% of lung fields; 3, 34 to 66% lung fields, and 4, >66% lung fields (*30*). (C) to (F) show representative histopathology images for vehicle control in (C) and (E) and compound ***6j*** treatment in (D) and (F) at 40× [(C) and (D)] or 100× [(E) and (F)]. Asterisks indicate *P* < 0.01 by multiple *t* tests.

The lung pathology caused by MERS_MA_-CoV infection of hDPP4-KI mice resembles that seen in severe cases of human MERS-CoV infection, with diffuse alveolar damage, pulmonary edema, hyaline membrane formation, and infiltration of lymphocytes into the alveolar septa (*22*). A group of hDPP4-KI mice were infected with MERS_MA_-CoV and treated with compound ***6j*** or vehicle as a control starting at 1 dpi. Mouse lungs were collected for determination of virus load at 3 and 5 dpi and for histopathology at 6 dpi. Lung virus titers decreased in the ***6j***-treated mice compared to control mice at both 3 and 5 dpi (*P* < 0.01) (Fig. 4A). Edema in the lungs of the treated mice was reduced compared to control mice (*P* < 0.01) (Fig. 4B). Scores for hyaline membrane formation were reduced in ***6j***-treated mice but were not statistically different from control mice. MERS_MA_-CoV–infected hDPP4-KI mice treated with vehicle showed patches in lung tissue variably composed of cellular inflammation, vascular congestion, and atelectasis (Fig. 4, C and E). The airways of these animals were generally intact, with only scattered, uncommon sloughed cells (Fig. 4, C and E). In some lungs from these mice, lymphatic vessels were filled with degenerative cells and cellular debris (Fig. 4, C and E). Alveolar edema was detected in some lung tissue sections (Fig. 4, C and E). In contrast, there were few observed lesions in the lungs of MERS_MA_-CoV–infected hDPP4-KI mice treated with compound ***6j*** starting at 1 dpi (Fig. 4, D and F).

## DISCUSSION

There are currently no approved vaccines or small-molecule therapeutics for the treatment of MERS-CoV, SARS-CoV, or SARS-CoV-2 infection. However, numerous preventive and therapeutic options are under development (*3*–*7*). The most clinically advanced antiviral compound with a broad spectrum of activity is remdesivir (GS-5734). This nucleoside analog was originally developed as an antiviral drug against Ebola virus and has been shown to be effective against both MERS-CoV and SARS-CoV in cell culture assays and in animal models of coronavirus infection (*23*–*26*). Prophylactic treatment or early therapeutic treatment of infected mice with remdesivir reduced MERS-CoV– or SARS-CoV–mediated weight loss and decreased lung virus titers and lung injury scores compared to those of vehicle-treated animals (*23*, *26*). Remdesvir also showed potent activity against SARS-CoV-2 in cell culture assays and animal models (*27*) and was recently issued an emergency use authorization by the U.S. Food and Drug Administration as an investigational antiviral drug for COVID-19. Another nucleoside analog, EIDD-2801, which is a broad-spectrum inhibitor against multiple viruses including influenza viruses, was also shown to be effective against MERS-CoV and SARS-CoV in mouse models (*28*).

Our group has been engaged in the discovery of broad-spectrum inhibitors targeting the 3CLpro of multiple human and animal coronaviruses. We initially generated dipeptidyl and tripeptidyl series of compounds (*29*) and observed that the dipeptidyl compound series had superior pharmacokinetic (PK) profiles compared to the tripeptidyl compound series (*20*). A representative compound of the dipeptidyl series is GC376, which is currently in clinical development for FIP in cats and COVID-19. The PK characteristics of multiple dipeptidyl compounds similar to compound ***6j***, including GC376, were examined through an intraperitoneal or subcutaneous administration in animals, including mice. It was determined that their maximum plasma concentration (C_max_) values were >100-fold of the EC_50_ for the target virus and that the elimination half-life (*T*_1/2_) was 3 to 5 hours. The in vivo efficacy of GC376 against mice infected with MHV or murine norovirus has been demonstrated (*11*, *15*).

Inspection of previously obtained crystal structures of the dipeptidyl compounds in a complex with coronavirus 3CLpro (*17*, *19*) revealed the potential to achieve enhanced binding interactions with the S_4_ subsite by introducing diverse functionalities at the cap position in the inhibitors. In the current study, a new dipeptidyl series focusing on the design of structural variants in the cap substructure were synthesized and evaluated for their activity against coronavirus 3CLpro. The EC_50_ of GC376 against MERS-CoV was determined to be ~1 μM. One of our goals was to generate compounds with near or below 0.1 μM potency against MERS-CoV and other target coronaviruses. All synthesized compounds displayed varying degrees of inhibitory activity against multiple coronaviruses in the FRET enzyme assay and cell-based assays. Among these compounds, ***6e*** showed the most potent antiviral activity against SARS-CoV-2 3CLpro in a FRET enzyme assay (IC_50_, 0.17 μM) and cell-based assays (EC_50_, 0.15 μM), and ***6j*** showed the most potent antiviral activity against MERS-CoV with an EC_50_ value of 0.04 μM (Table 2).

It was previously demonstrated that optimal potency is attained when the P_1_ and P_2_ residues are a glutamine surrogate and Leu, respectively, and that replacement of the P_2_ Leu with a cyclohexylalanine is inimical to potency (*17*, *18*). This is clearly evident when comparing the relative potencies of ***6h*** and ***7h*** versus ***6i*** and ***7i*** (Table 1 and table S1). Furthermore, compounds with Leu at the P_2_ position showed higher CC_50_ values compared to those with cyclohexylalanine at P_2_ (table S1). X-ray crystallography confirmed the mechanism of action of the inhibitors, which involves formation of a covalent bond between the active site cysteine and the carbonyl carbon of the aldehyde. X-ray crystallography also identified the structural determinants associated with binding, accounting for the observed differences in potency. The high-resolution cocrystal structures of 3CLpro inhibitors ***7j*** and ***7i*** with MERS-CoV 3CLpro also confirmed that the difference in activity arose from the loss of a H-bond with Gln^192^ and the loss of two additional H-bonds from the displacement of Gln^167^ and Phe^143^ with ***7i*** (fig. S2A). The nature of the interaction of ***7j*** with the S_4_ subsite is unique among the compounds examined and provides strong support for our approach, vis-à-vis our focus on the cap position for enhancing binding affinity and potency. Compound ***7j*** was cocrystallized with MERS-CoV, SARS-CoV, and SARS-CoV-2 3CLpro. Superposition of compound ***7j*** with these 3CLpro enzymes revealed a similar binding mode among all three proteases. However, a key difference lies with the different conformation adopted by the difluorocyclohexyl ring in the MERS-CoV 3CLpro S_4_ subsite, enabling it to engage in additional H-bond binding interactions (Fig. 2E and fig. S4). Compound ***7j*** had a moderately lower potency against SARS-CoV 3CLpro and SARS-CoV-2 3CLpro compared to MERS-CoV 3CLpro in the FRET enzyme assay, suggesting that moieties forming H-bonds that were accommodated at the S_4_ subsite had an important impact on potency. The barrier to the development of drug resistance increases when an inhibitor engages in H-bond interactions with the backbone of the 3CLpro.

We used a robust mouse model of MERS-CoV infection (*30*–*32*) to evaluate the efficacy of compounds ***6j*** and ***6h***. hDPP4-KI mice expressing human dipeptidylpeptidase 4 were infected with a mouse-adapted MERS-CoV virus (MERS_MA_-CoV). The infected mice develop fatal lung disease with severe inflammation and weight loss (*32*). Furthermore, the lung pathology caused by MERS_MA_-CoV infection of the hDPP4-KI mice closely resembles that of severe human MERS-CoV infection and is characterized by diffuse alveolar damage, pulmonary edema, hyaline membrane formation, and infiltration of lymphocytes into the alveolar septa (*22*). In the current study, we demonstrated that survival rates in this mouse model were higher with ***6j*** treatment compared to ***6h*** treatment (Fig. 3B). Compounds ***6j*** and ***6h*** share a near-identical structure except for the extra methylene group present in compound ***6j***. Compound ***6h*** showed potent anti-3CLpro activity, whereas the antiviral activity of compound ***6h*** in cell culture was lower than that of compound ***6j*** (Tables 1 and 2), which may have been the reason for its diminished therapeutic efficacy in the mouse model (Fig. 3B).

Our findings indicate that therapeutic treatment of infected mice with compound ***6j*** was associated with a reduction in lung viral load and lung pathology (Fig. 4). Moreover, treatment of mice with ***6j*** at 1 dpi resulted in the survival of infected mice, whereas delaying treatment initiation until 3 dpi resulted in decreased survival. Overall, mouse survival was markedly increased only when ***6j*** was given to mice at 1 dpi (*P* < 0.05; Fig. 3, D and E). Treatment with compound ***6j*** starting at 2 dpi resulted in moderately increased survival of infected mice, but this was not statistically significant (*P* > 0.05). These results emphasize the importance of early therapeutic intervention in attaining a positive clinical outcome.

Earlier studies from our group showed that GC376 can cure fatal feline coronavirus disease in cats (*20*, *21*), demonstrating that a specific coronavirus protease inhibitor can be effective therapeutically against coronavirus disease in a natural host. The MERS-CoV mouse model used here provides proof of principle regarding the therapeutic potential of our protease inhibitors for treating severe human respiratory coronavirus disease. Limitations of the current study include differences in host receptor usage, mortality, and transmissibility between MERS-CoV and SARS-CoV-2. Thus, further evaluation of our protease inhibitors in mice, hamsters, or nonhuman primates experimentally infected with SARS-CoV-2 will be crucial to assess these inhibitors as potential therapeutic options for COVID-19.

Our study showed that these compounds were broadly active against the 3CLpro of several coronaviruses, with compound ***6j*** displaying the highest activity against MERS-CoV and compound ***6e*** displaying the highest activity against SARS-CoV-2. Clinical efficacy is influenced by many factors, including drug bioavailability, PK, metabolism, and the chemical stability of a compound. This poses a major challenge with respect to reliably predicting whether the difference in potency against different coronaviruses in assays in vitro can be translated to differences in clinical efficacy. Therefore, further research is needed to establish whether one protease inhibitor can be an effective therapeutic for both MERS-CoV and SARS-CoV-2 infections in humans. The results from our previous and current studies suggest that the dipeptidyl compound series can serve as a platform suitable for the structure-guided design of one or more inhibitors against highly virulent human coronaviruses. We have generated potent inhibitors of the 3CLpro of several coronaviruses, including SARS-CoV-2, and tested their efficacy in cultured cells and primary human airway epithelial cells. Furthermore, we have demonstrated proof-of-concept therapeutic efficacy for one 3CLpro inhibitor ***6j*** in hDPP4-KI mice infected with MERS_MA_-CoV. Our study lays the foundation for advancing this compound series further along the drug development pipeline.

## MATERIALS AND METHODS

### Study design

The goal of this study was to evaluate the efficacy of 3CLpro inhibitors against human coronaviruses, including SARS-CoV-2, in a FRET enzyme assay and cell culture assays, as well as in a mouse model of MERS-CoV infection. Initial antiviral screening was performed with recombinant 3CLpro from SARS-CoV, MERS-CoV, and SARS-CoV-2 in the FRET enzyme assay. Antiviral activity was then assessed in cultured Huh-7 cells infected with MERS-CoV and Vero E6 cells infected with SARS-CoV-2. Selected 3CLpro inhibitors were further examined using x-ray cocrystallization with MERS-CoV, SARS-CoV, and SARS-CoV-2 3CLpro to elucidate the mechanism of action and identify the structural determinants of potency. Last, two selected compounds were evaluated for in vivo efficacy in a mouse model of MERS-CoV infection (hDPP4-KI mice expressing human dipeptidylpeptidase 4 infected with a mouse-adapted MERS-CoV). Age- and sex-matched mice were randomly assigned into various groups for virus infection and treatment studies. Microscopic analysis of lung lesions was conducted in a blinded manner; other experiments were not blinded. No mice were excluded from analysis.

In vivo studies were performed in animal biosafety level 3 facilities at the University of Iowa. All experiments were conducted under protocols approved by the Institutional Animal Care and Use Committee at the University of Iowa according to guidelines set by the Association for the Assessment and Accreditation of Laboratory Animal Care and the U.S. Department of Agriculture.

The studies with MERS-CoV and SARS-CoV-2 were performed in biosafety level 3 facilities at the University of Iowa. All experiments were conducted under protocols approved by the Institutional Biosafety Committee at the University of Iowa according to guidelines set by the Biosafety in Microbiological and Biomedical Laboratories, the U.S. Department of Health and Human Services, the U.S. Public Health Service, the U.S. Centers for Disease Control and Prevention, and the National Institutes of Health.

### Synthesis of 3CL protease inhibitors

Compounds ***6a*** to ***6k*** and ***7a*** to ***7k*** were readily synthesized as illustrated in Fig. 1 and are listed in Table 1 and table S1. Briefly, the alcohol inputs were reacted with l-Leucine isocyanate methyl ester or l-cyclohexylalanine isocyanate methyl ester to yield dipeptides “***2***”, which were then hydrolyzed to the corresponding acids with lithium hydroxide in aqueous tetrahydrofuran. The subsequent coupling of the acids to glutamine surrogate methyl ester “***8***” (*33*, *34*) furnished compounds “***4***”. Lithium borohydride reduction yielded alcohols “***5***”, which were then oxidized to the corresponding aldehydes “***6***” with Dess-Martin periodinane reagent. The bisulfite adducts “***7***” were generated by treatment with sodium bisulfite in aqueous ethanol and ethyl acetate (*35*).

### FRET enzyme assay

The expression and purification of the 3CLpro of MERS-CoV and SARS-CoV were performed by a standard method described previously by our lab (*11*, *19*, *20*). We also cloned and expressed the 3CLpro of SARS-CoV-2. The codon-optimized complementary DNA (cDNA) of the full length of 3CLpro of SARS-CoV-2 (GenBank accession number MN908947.3) fused with sequences encoding six histidine at the N terminus was synthesized by Integrated DNA (Coralville, IA). The synthesized gene was subcloned into the pET-28a(+) vector. The expression and purification of SARS-CoV-2 3CLpro were conducted after a standard procedure described by our lab (*19*). Briefly, stock solutions of compounds ***6a*** to ***6k*** and ***7a*** to ***7k*** were prepared in dimethyl sulfoxide (DMSO) and diluted in assay buffer, which was composed of 20 mM Hepes buffer (pH 8) containing NaCl (200 mM), EDTA (0.4 mM), glycerol (60%), and 6 mM dithiothreitol (DTT). The protease (3CLpro of MERS-CoV, SARS-CoV, or SARS-CoV-2) was mixed with serial dilutions of each compound or with DMSO in 25 μl of assay buffer and incubated at 37°C for 30 min (MERS-CoV) or at room temperature for 1 hour (SARS-CoV and SARS-CoV-2), followed by the addition of 25 μl of assay buffer containing substrate (FAM-SAVLQ/SG-QXL 520, AnaSpec, Fremont, CA). The substrate was derived from the cleavage sites on the viral polyproteins of SARS-CoV. Fluorescence readings were obtained using an excitation wavelength of 480 nm and an emission wavelength of 520 nm on a fluorescence microplate reader (FLx800, BioTek, Winooski, VT) 1 hour after the addition of substrate. Relative fluorescence units (RFU) were determined by subtracting background values (substrate-containing well without protease) from the raw fluorescence values, as described previously (*19*). The dose-dependent FRET inhibition curves were fitted with a variable slope by using GraphPad Prism software (GraphPad, La Jolla, CA) to determine the IC_50_ values of the compounds.

### Antiviral cell-based assays

Some compounds in the ***6a*** to ***6k*** and ***7a*** to ***7k*** series were also investigated for their antiviral activity against the replication of MERS-CoV, FIPV, or MHV-1 in Huh-7, CRFK, or L929 cells, respectively (*19*). Briefly, medium containing DMSO (<0.1%) or each compound (up to 100 μM) was added to confluent cells, which were immediately infected with viruses at a multiplicity of infection (MOI) of 0.01. After incubation of the cells at 37°C for 24 hours, viral titers were determined with the median tissure culture infectious dose (TCID_50_) method (FIPV or MHV) with the CRFK or L929 cells or plaque assay with Vero81 cells (MERS-CoV). For SARS-CoV-2, confluent Vero E6 cells were inoculated with ~50 to 100 PFU per well, and medium containing various concentrations of each compound and agar was applied to the cells. After 48 to 72 hours, plaques in each well were counted. EC_50_ values were determined by GraphPad Prism software using a variable slope (GraphPad, La Jolla, CA) (*19*). To confirm that these inhibitors also inhibit SARS-CoV-2 in primary human cells, differentiated human airway epithelial cells from three donors were used as previously described (*36*, *37*). Two compounds (***6j*** and ***6e***) were tested for their antiviral effects against SARS-CoV-2. Briefly, airway epithelial cells were washed with phosphate-buffered saline (PBS), and SARS-CoV-2 was inoculated at a MOI of 0.1 for 1 hour. After the inoculum was removed, media containing ***6j*** (2 μM) or ***6e*** (0.5 μM) was added. After 48 hours, cells were subjected to a freeze/thaw cycle, and virus titers were determined by plaque assay on Vero E6 cells.

### Measurement of cytotoxicity

The 50% cytotoxic concentration (CC_50_) for compounds ***6a*** to ***6k*** and ***7a*** to ***7k*** was determined in Huh-7, CRFK, or L929 cells. Confluent cells grown in 96-well plates were incubated with various concentrations (1 to 100 μM) of each compound for 72 hours. Cell cytotoxicity was measured by a CytoTox 96 nonradioactive cytotoxicity assay kit (Promega, Madison, WI), and the CC_50_ values were calculated using a variable slope by GraphPad Prism software. The in vitro therapeutic index was calculated by dividing the CC_50_ by the EC_50._

### Protein purification, crystallization, and data collection in x-ray crystallographic studies

MERS-CoV 3CLpro and SARS-CoV 3CLpro were purified as described previously (*17*, *19*). An *Escherichia coli* codon-optimized construct encoding residues Ser^3264^ to Phe^3568^ of the orf1ab polyprotein (SARS-CoV-2 3CLpro, Genebank accession number QHD43415.1) was cloned into a pET His6 Sumo TEV LIC cloning vector (2S-T, Addgene). Expression and initial Ni-column purification were performed as described for MERS-CoV 3CLpro and SARS-CoV 3CLpro. The Small Ubiquitin-like Modifier protein (SUMO) fusion elution fractions of SARS-CoV-2 were dialyzed against 20 mM tris (pH 8.0) and 100 mM NaCl and treated with the tobacco etch virus (TEV) protease (1:10, w/w) overnight. This mixture was loaded onto a 5-ml HisTrap HP column (GE Healthcare) equilibrated with 20 mM tris (pH 8.0) and 100 mM NaCl and eluted with 20 mM tris (pH 8.0), 100 mM NaCl, and 500 mM imidazole using an ÄKTA Pure fast protein liquid chromatography (FPLC). The flow-through fractions containing SARS-CoV-2 3CLpro without the SUMO fusion were loaded onto a Superdex 75 10/300 GL size exclusion column equilibrated with 20 mM tris (pH 8.0) and 100 mM NaCl. The fractions were pooled and concentrated to 9.6 mg/ml for crystallization screening. Note that four residues from cloning (Ser-Asn-Ile-Gly) remain at the N terminus after treatment with TEV protease.

Purified MERS-CoV 3CLpro, SARS-CoV 3CLpro, and SARS-CoV-2 3CLpro in 100 mM NaCl and 20 mM tris (pH 8.0) were concentrated to 10.6 mg/ml (0.3 mM), 22 mg/ml (0.64 mM), and 9.6 mg/ml (0.28 mM), respectively, for crystallization screening. All crystallization experiments were set up using an NT8 drop-setting robot (Formulatrix Inc.) and UVXPO MRC (Molecular Dimensions) sitting-drop vapor diffusion plates at 18°C. One hundred nanoliters of protein and 100 nl of crystallization solution were dispensed and equilibrated against 50 μl of the latter. Stock solutions (100 mM) of compounds ***6b***, ***6d***, ***6g***, ***6h***, ***7i***, and ***7j*** were prepared in DMSO, and complexes were generated by mixing 1 μl of the ligand (2 mM) with 49 μl (0.29 mM) of the protease and incubating on ice for 1 hour. Crystals of the MERS-CoV 3CLpro inhibitor complexes were obtained from the following conditions: compounds ***6b***, ***6d***, ***6g***, and ***6h***: ProPlex screen (Molecular Dimensions) condition E2 [8% (w/v) poly(ethylene glycol) (PEG) 8000 and 100 mM sodium citrate (pH 5.0)], compound ***7i***: ProPlex screen (Molecular Dimensions) condition B8 [15% (w/v) PEG 4000, 100 mM sodium citrate (pH 5.0), and 100 mM magnesium chloride], and compound ***7j***: Index HT screen (Hampton Research) condition F6 [25% (w/v) PEG 3350, 100 mM bis-tris (pH 5.5), and 200 mM ammonium sulfate]. Crystals of the SARS-CoV 3CLpro complex with compound ***7j*** were obtained from the Index HT screen (Hampton Research) condition H8 [15% (w/v) PEG 3350 and 100 mM magnesium formate]. Crystals of the SARS-CoV-2 3CLpro complex with compound ***7j*** were obtained in 1 to 2 days from the PACT HT screen (Molecular Dimensions) condition D7 [20% (w/v) PEG 6000, 100 mM tris (pH 8.0), and 200 mM NaCl]. Samples were transferred to a fresh drop containing 80% crystallant and 20% (v/v) PEG 200 before storing in liquid nitrogen. X-ray diffraction data were collected at the Advanced Photon Source beamline 17-ID using a Dectris PILATUS 6M (MERS-CoV 3CLpro and SARS-CoV 3CLpro) and Dectris EIGER2 X 9M (SARS-CoV-2 3CLpro) pixel array detector.

### Solution and refinement of crystal structures

Intensities were integrated using XDS (*38*, *39*) using autoPROC (*40*), and the Laue class analysis and data scaling were performed with Aimless (*41*). Structure solution was conducted by molecular replacement with Phaser (*42*) using a previously determined structure of MERS 3CLpro [Protein Data Bank (PDB): 5WKK (*17*)] and SARS-CoV 3CLpro [PDB: 1Q2W (*43*)] and SARS-CoV-2 3CLpro [PDB: 6LU7(*44*)] as the search models. Structure refinement and manual model building were conducted with Phenix (*45*) and Coot (*46*), respectively. Disordered side chains were truncated to the point for which electron density could be observed. Structure validation was conducted with MolProbity (*47*), and figures were prepared using the CCP4MG package (*48*). Crystallographic data are provided in table S2.

### Therapeutic treatment in a mouse model of MERS-CoV infection

The two best compounds (***6j*** and ***6h***) in the series were examined for their in vivo efficacy using 10-week-old male hDPP4-KI mice infected with MERS_MA_-CoV (*30*). In the first study, animals were divided into three groups (*n* = 5 to 6) and were lightly anesthetized with ketamine/xylazine and infected with 50 μl of 750 PFU MERS_MA_-CoV via intranasal inoculation. Compound ***6j*** or ***6h*** were formulated in 10% ethanol and 90% PEG400 and given to mice from 1 to 10 dpi at 50 mg/kg per day (once per day) via intraperitoneal administration. The control mice received vehicle. Animals were weighed daily and monitored for 15 days. Animals were euthanized when an animal lost 30% of initial weight or at 15 dpi.

In the next study, treatment with compound ***6j*** was delayed up to 3 dpi to determine the impact of delayed treatment on mouse survival. Animals were divided into five groups (*n* = 5), and compound ***6j*** (50 mg/kg per day, once per day) was administered to mice starting at 1, 2, or 3 days after virus challenge (1, 2, or 3 dpi, respectively) until 10 dpi. Mice were monitored for weight loss and survival as described above for 15 days after virus challenge. As controls, vehicle (10% ethanol and 90% PEG400) was administered equivalently to the experimental compound, or animals received no treatment (untreated). The third study was conducted to assess the effects of therapeutic treatment of compound ***6j*** in the lungs. For lung harvest and virus titration, animals were divided into three groups (*n* = 4) of mice, and compound ***6j*** (50 mg/kg per day, once per day) or vehicle was administered to mice starting at 1 dpi until euthanasia. Animals were euthanized at 3 or 5 dpi, and lungs were removed aseptically, disassociated with a manual homogenizer in 1× PBS, briefly centrifuged, and supernatants removed. Samples were titered on Vero81 cells as reported elsewhere (*49*). For lung histopathology analyses, animals were divided into two groups (*n* = 5), and compound ***6j*** (50 mg/kg per day, once per day) or vehicle was administered to mice starting at 1 dpi for 5 days. Mice were euthanized at 6 dpi, lungs were fixed with 10% formalin, and hematoxylin and eosin–stained tissues were examined by a veterinary pathologist using the postexamination method of masking (*50*). Briefly, tissues were scored in an ordinal manner for edema and hyaline membrane formation using the following scale: 0, none; 1, rare (<5 alveoli); 2, <33% of lung fields; 3, 34 to 66% lung fields; and 4, >66% lung fields (*30*).

### Statistical analysis

The analysis of survival curves in groups was performed using a log-rank (Mantel-Cox) test and a Gehan-Breslow-Wilcoxon test using GraphPad Prism software (San Diego, CA). Log-transformed viral titers in the lungs and lung edema and hyaline membrane formation in groups of mice were analyzed with multiple *t* tests using GraphPad Prism software.

## SUPPLEMENTARY MATERIALS

stm.sciencemag.org/cgi/content/full/12/557/eabc5332/DC1 Materials and Methods Fig. S1. X-ray cocrystal structures of compounds with coronavirus 3CLpro showing the *F*_o_-*F*_c_ omit map. Fig. S2. X-ray cocrystal structures of compounds with coronavirus 3CLpro showing hydrogen bond interactions. Fig. S3. X-ray cocrystal structures of compounds with coronavirus 3CLpro showing electrostatic surface representation. Fig. S4. X-ray cocrystal structures of comparative binding of ***7j*** with the 3CLpro of MERS CoV, SARS-CoV, and SARS-CoV-2. Table S1. Compound IC_50_ in the FRET enzyme assay and CC_50_ in a cell culture assay for MERS-CoV 3CLpro. Table S2. Cocrystal structure data for compounds with the 3CLpro of MERS CoV and SARS CoV. Data file S1. Individual-level data for all figures and tables. References (*51*–*55*)

## Appendix

View/request a protocol for this paper from *Bio-protocol*.
